# Letter from the Editor-in-Chief

**DOI:** 10.19102/icrm.2017.080307

**Published:** 2017-03-15

**Authors:** Moussa Mansour


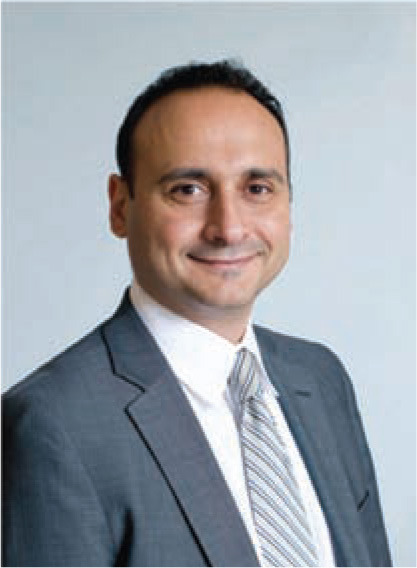


Dear Readers,

As the number of procedures and indications of catheter ablation for cardiac arrhythmias continue to expand, it has become increasingly common to perform ablation in patients with implantable cardiac devices. However, there are specific considerations that need to be taken into account in this group of patients, in order to avoid complications and ensure a successful outcome of the ablation procedure. This issue of the *Journal* contains an interesting article by Darrat et al. titled “The Effects of Catheter Ablation on Permanent Pacemakers and Implantable Cardiac Defibrillators.” In this article, the authors review the potential interactions between cardiac implantable devices and catheter ablation, including the effects on the pulse generator and lead dislodgment, as well as the effects of ablation performed close to or in direct contact with a device lead. In addition, the authors also describe their peri-ablation protocol for device management in these patients.

This article is important for two reasons. First, it defines in detail the mechanisms of potential complications that one can encounter in patients with devices during ablation. Second, it highlights a rapidly growing trend of performing ablation for various cardiac arrhythmias in these patients. Central to this group are patients with cardiac resynchronization therapy (CRT) devices, who not only have multiple pacing and defibrillation leads, but who also have comorbidities that typically render the ablation procedure longer and more complicated. This in turn increases the chance of unfavorable interaction between ablation and device technologies.

A sign of the increased interest in ablation in patients with cardiac devices is the emergence of clinical trials investigating this treatment modality. One important study is ABLATE-CRT (Catheter Ablation of Arrhythmias to Improve CRT Response). The study, designed by Dr. Dhanunjaya Lakkireddy, is expected to start enrolling patients soon. It tests the hypothesis that ablation improves the rate of CRT response in patients with atrial fibrillation/flutter, supraventricular tachycardia, premature ventricular contractions, and/or ventricular tachycardia. Subjects will be randomized to either undergo ablation or conventional treatment. As ABLATE-CRT and other similar clinical trial emerge, it is expected to become increasingly important to also develop algorithms and strategies that help reduce the complications resulting from the interaction between ablation and device technologies.

I hope that you enjoy reading this issue of the *Journal.*

Sincerely,


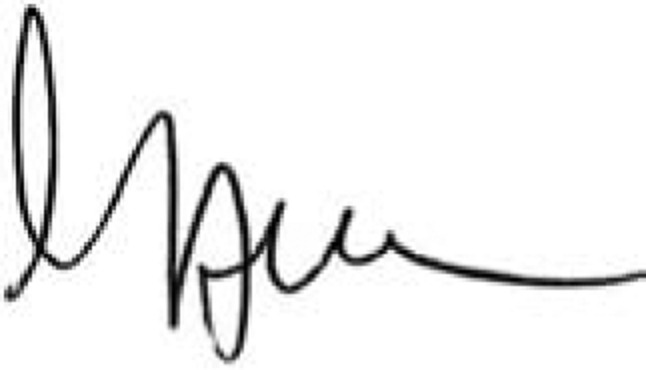


Moussa Mansour, MD, FHRS, FACC

Editor-in-Chief

The Journal of Innovations in Cardiac Rhythm Management

MMansour@InnovationsInCRM.com

Director, Cardiac Electrophysiology Laboratory

Director, Atrial Fibrillation Program

Massachusetts General Hospital

Boston, MA 02114

